# Next-Generation Biomarkers in Multiple Myeloma: Understanding the Molecular Basis for Potential Use in Diagnosis and Prognosis

**DOI:** 10.3390/ijms22147470

**Published:** 2021-07-13

**Authors:** Amro M. Soliman, Srijit Das, Seong Lin Teoh

**Affiliations:** 1 Department of Biological Sciences—Physiology, Cell and Developmental Biology, University of Alberta, Edmonton, AB T6G 2R3, Canada; amsherif@ualberta.ca; 2 Department of Human & Clinical Anatomy, College of Medicine & Health Sciences, Sultan Qaboos University, P.O. Box 35, Al-Khoud, Muscat 123, Oman; 3 Department of Anatomy, Faculty of Medicine, Universiti Kebangsaan Malaysia Medical Centre, Kuala Lumpur 56000, Malaysia

**Keywords:** multiple myeloma, diagnostic markers, prognostic markers, miRNAs, angiogenic markers, liquid biopsy, telomeres, proteomics

## Abstract

Multiple myeloma (MM) is considered to be the second most common blood malignancy and it is characterized by abnormal proliferation and an accumulation of malignant plasma cells in the bone marrow. Although the currently utilized markers in the diagnosis and assessment of MM are showing promising results, the incidence and mortality rate of the disease are still high. Therefore, exploring and developing better diagnostic or prognostic biomarkers have drawn global interest. In the present review, we highlight some of the recently reported and investigated novel biomarkers that have great potentials as diagnostic and/or prognostic tools in MM. These biomarkers include angiogenic markers, miRNAs as well as proteomic and immunological biomarkers. Moreover, we present some of the advanced methodologies that could be utilized in the early and competent diagnosis of MM. The present review also focuses on understanding the molecular concepts and pathways involved in these biomarkers in order to validate and efficiently utilize them. The present review may also help in identifying areas of improvement for better diagnosis and superior outcomes of MM.

## 1. Introduction

Multiple myeloma (MM), also known as plasma cell myeloma, is a clonal plasma cell (PC) malignancy. Subsequent to PCs’ maturation in lymph nodes, they migrate to the bone marrow (BM), where they undergo malignant changes to develop the disease [1,2]. The abnormal proliferation of PCs leads to the extensive involvement of the skeletal system, with osteopenia/osteolytic lesions, anemia, hypercalcemia and soft tissue plasmacytomas [3,4].

Being the second most common blood cancer, MM accounts for 13% of all hematologic malignancies worldwide [5,6,7]. In the United States of America, more than 230,000 cases of MM were reported between 2011 and 2016 [5,8]. The overall survival (OS) of MM patients was 30 months and could be extended to 46 months with Thalidomide treatment, and 48 months by BM transplantation [9]. More importantly, the survival rate is lower in patients with a higher ISS stage, suggesting that an early and competent diagnosis of MM may promote the survival rate [10].

MM patients are usually preceded by pre-malignant asymptomatic stages, recognized as the monoclonal gammopathy of undetermined significance (MGUS) or smoldering MM (SMM) that progresses towards symptomatic medullary and extramedullary MM [11]. Although there are several established biomarkers that are currently utilized for diagnosis of MM, assessment of tumor burden and risk-stratifying patients [2], the incidence of MM cases is progressively increasing. Therefore, researchers are investigating the development of novel biomarkers that could help in better diagnosis, prognosis, or treatment. Characterizing effective, sensitive and specific diagnostic and/or prognostic biomarkers is essential to detect the disease at early stages, thus effectively combating the malignancy. Numerous studies are currently investigating several novel biomarkers that can be used in the diagnosis and prognosis of myeloma disease. Fortunately, advances in understanding the biology of the disease opened the door to the development of new diagnostic and prognostic approaches. A thorough comprehension of the pathogenesis of MM and the molecular concepts of these novel biomarkers are vital for their validation and proper utilization.

In the present review, we comprehensively explain the recently reported biomarkers that can be utilized for the diagnosis, prognosis and management of MM, along with their underlying molecular mechanisms. Furthermore, we highlight the limitations and future areas of improvements of these biomarkers.

## 2. Molecular Pathogenesis and Current Biomarkers of MM

**Figure 1:**
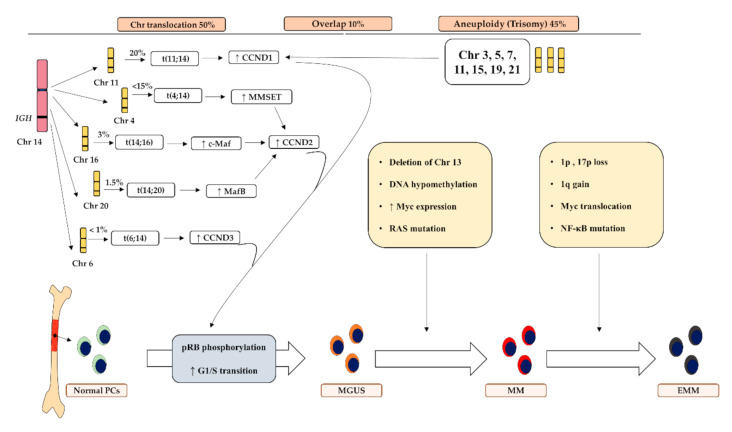
Molecular pathogenesis and underlying cytogenic alterations associated with MM development. Chromosomal translocations and aneuploidy disrupt cyclin D genes that in turn enhance G1/S transition of the cell cycle to induce abnormal cellular proliferation in plasma cells to develop MGUS. Other genetic mutations were found to be associated with the transformation of the disease towards MM and EMM. IGH; Immunoglobulin H, Chr: chromosome, PCs: plasma cells; MGUS: monoclonal gammopathy of undetermined significance, SMM: smoldering multiple myeloma, EMM: extramedullary multiple myeloma, CCDN: cyclin D, MMSET: multiple myeloma SET domain, NF-κB: nuclear factor kappa-light-chain-enhancer of activated B cells, BM: bone marrow.

Although the exact cause of MM is still undetermined, several genetic mutations are related to MM development. The intraclonal genetic heterogeneity of PCs drives the progression of the disease [12]. Particular cytogenetic variations correlated with the development of MGUS, while other oncogenes and mutations were linked to the progression of MGUS to MM or extramedullary MM (Figure 1) [13,14]. Furthermore, cytogenetic alternations were found to greatly impact the treatment outcome, drug resistance and prognosis of MM [14].

Normally, PCs remain in an arrested state by holding their cell cycle at G0/G1 phase in the BM unless activated by a microbial challenge. To develop MM, these cells acquire abnormal growth and proliferation capabilities. This is where the genetic alterations play a role by dysregulating critical pathways involved in controlling G0/G1 transition [15]. Chromosomal translocation, aneuploidy and chromosomal deletion contribute significantly to MM development via targeting the cyclin D (CCDN) family, a crucial regulator of G0/G1, through several pathways (Figure 1). The most common translocation in MM is t(11;14)(q13;q32), which was detected in 20% of MM patients and involved the translocation of the immunoglobulin heavy chain (IgH) gene locus [16]. The t(11;14) is also associated with a poorer outcome in MM patients [16]. Additionally, translocation of the immunoglobulin lambda (IgL) gene locus was also reported in 10% of MM patients, and also served as an indicator of poor prognosis [17].

The evolvement of MGUS to MM was shown to be associated with the up-regulation of oncogenes such as Myc and Ras [18,19], chromosome deletion and other factors [20]. The activation of Myc was suggested to be induced by super enhancer-mediated hyperactivation of transcription that could be experimentally blocked via small molecular inhibitors, thus representing a potential drug target [21,22]. Other contributors encompass DNA hypomethylation, which causes genome instability [20,23].

**Figure 2:**
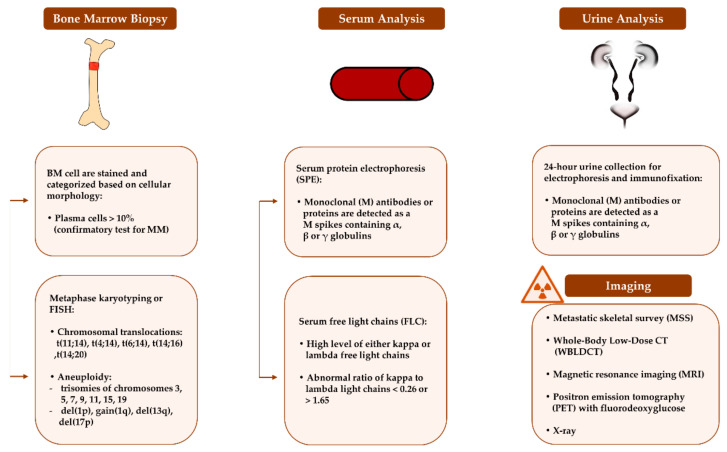
Biomarkers that are currently utilized for diagnosis and risk-stratifying of multiple myeloma.

Developing and establishing diagnostic tools is increasingly becoming critical for managing MM. Currently, several biomarkers are deployed for diagnosis and prognosis of the disease (Figure 2). The levels of these biomarkers are evaluated based on diagnostic procedures such as BM biopsy, serum analysis, 24-hour urine analysis, metaphase karyotyping, Fluorescence In Situ Hybridization (FISH) and imaging [24]. The assessment of monoclonal protein (M protein), the abnormal immunoglobulin (Ig) produced by malignant PCs and the free light chain (FLC) level in the serum and urine of MM patients is critical for diagnosis. Similarly, detecting chromosomal abnormalities and osteolytic bone lesions help in risk-stratifying of MM cases.

**Figure 3:**
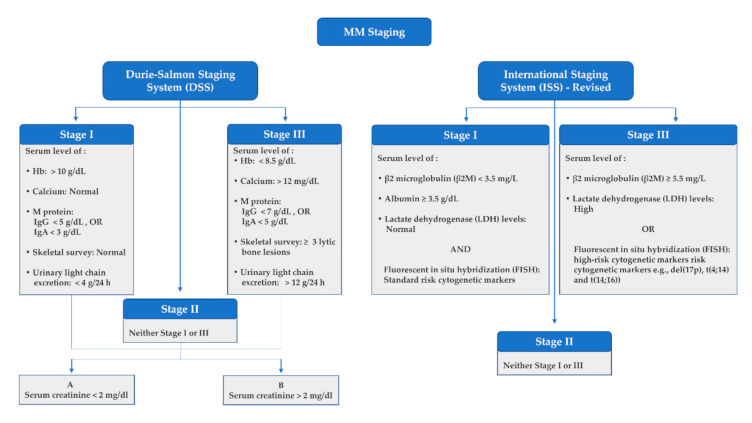
Multiple myeloma staging systems: Durie-Salmon PLUS System (DSS) and revised International Staging System (ISS).

Guidelines were developed for differentiating the phases of MM concerning early asymptomatic phases of the disease, e.g., MGUS, in addition to the more advanced, symptomatic phases that comprise medullary and extramedullary MM. The International Myeloma Working Group (IMWG) presented diagnostic benchmarks for MM and its differential phases, including IgM MGUS, Non-IgM MGUS, Light chain MGUS and SMM [25]. These diagnostic criteria considered several biomarkers that involved the serum or urinary levels of the M protein and evidence of end-organ failure that was judged by serum levels of calcium, hemoglobin (Hb) and creatinine [25]. Other established guidelines for MM staging incorporated two systems, i.e., the Durie-Salmon PLUS System (DSS) and the revised International Staging System (ISS) (Figure 3). Both the DSS and the ISS are primarily used for classifying the progression and advancement of the MM to determine the most suitable and effective treatment protocols as well as to assess the median survival. These staging systems also depend mainly on the serum or urine levels of specific biomarkers in addition to allied cytogenic alterations [26,27].

Both IMWG criteria and staging systems are crucial for the management of MM; however, they are reliant on diagnostic and prognostic biomarkers such as M protein and β2-microglobulin levels. Therefore, the stability and reproducibility of these diagnostic and staging guidelines are liable to the sensitivity and consistency of these biomarkers. M-protein, for example, is not detected in about 18% of MM cases via serum protein electrophoresis [28], with nearly 3% of MM patients having no reportable trace of M protein [29], resulting in misdiagnosis and inaccurate staging in those cases. Furthermore, β2-microglobulin levels could be affected by several factors, including kidney and liver diseases. As a result, several research groups are currently investigating the utilization of other novel biomarkers that can overcome the limitations associated with current diagnostic tools.

## 3. Novel Biomarkers for Diagnosis and Prognosis of MM

Due to the progressively increasing numbers of MM cases, despite the currently used diagnostic and prognostic biomarkers, several next-generation biomarkers are currently emerging that could enhance clinical management and improve the outcome of the disease. In the following sections, we report the recently reported biomarkers with their underlying molecular mechanisms. Moreover, we illustrate their potential and areas of improvement to establish these biomarkers in future guidelines for early diagnosis and accurate assessment of the disease.

### 3.1. Angiogenesis Markers

To maintain their expediated growth and cellular proliferation, malignancies promote new blood vessel formation, a process known as angiogenesis, to sufficiently supply cancer cells with oxygen and nutrients, and for waste disposal [30]. In cancer, this may involve the increased production of activators (pro-angiogenic factors) or loss of inhibitors (anti-angiogenic factors). Therefore, angiogenic markers are considered crucial tumor markers in various malignancies. Several pro-angiogenic factors were characterized in the past few decades. Hepatocyte growth factor (HGF), vascular endothelial growth factor (VEGF), fibroblast growth factor (FGF) and angiopoietins represent key pro-angiogenic signals. Among these signals, VEGF was reported to be over-expressed in several malignancies and it has been well studied as a potential therapeutic target [31,32,33]. In MM, VEGF, in addition to HGF, angiopoietins and JunB were up-regulated, where they demonstrated a potential diagnostic and prognostic marker [34,35,36]. The experimental down-regulation of VEGF by chemotherapeutic agents and herbal products was associated with a significant reduction in cellular proliferation and an increase in apoptotic myeloma cells [37,38,39]. Similarly, other pro-angiogenic factors such as angiopoietin-1, angiopoietin-2 and HGF expression were impeded by these therapeutic applications, suggesting their potential as prognostic biomarkers [38].

The molecular pathways regulating angiogenesis in MM include epidermal growth factor receptor (EGFR) and its ligand heparin-binding EGF-like growth factor (HB-EGF). The HB-EGF-EGFR pathway was found to promote endothelial cell proliferation in BM angiogenesis in vivo and in vitro [40]. Moreover, the high expression levels of EGFR and HB-EGF in MM cells, when compared with cells from MGUS patients, were associated with an increase in the percentage of MM PCs. Furthermore, inhibiting the HB-EGF–EGFR signaling pathway resulted in limiting the angiogenic ability of BM endothelial cells [40]. Other angiogenic pathways involve BM thrombopoietin (TPO), which was revealed to maintain and endorse angiogenesis in MM. Interestingly, the TPO level varies significantly at different phases of MM. For instance, BM and serum TPO increased remarkably with the progression from MGUS/SMM to MM, suggesting its utilization as a potential biomarker in MM diagnosis and prognosis [41]. TPO receptors are expressed in BM endothelial cells, where they are activated to trigger intracellular angiogenic signaling pathways that enhance cell migration and chemotaxis in vitro as well as up-regulating matrix metalloproteinase (MMP)-9 and MMP-2, disrupting the balance between the angiogenic/anti-angiogenic factors in the BM [41].

Mesenchymal stromal cells are non-hematopoietic multipotent cells that play an important role in MM development and progression via coordinating cellular migration and enhancing angiogenesis [42]. The stromal cells cultured with MM cell lines (U266/Lp-1) under hypoxic conditions were associated with a rise in α-smooth muscle actin, hypoxia-inducible factor (HIF)-2α and integrin-linked kinase proteins, indicating their role as potential angiogenic markers [43]. Interestingly, the inhibition of HIF-2α reduced both α-smooth muscle actin and integrin-linked kinase, resulting in attenuating angiogenesis in vitro. Mechanistically, the HIF-2α released by stromal cells promotes angiogenesis via increasing the attachment of Q-dot labeled cells and the excretion of angiogenic factors [43]. Along with the role of these angiogenic markers in the diagnosis/prognosis of MM, they represent possible drug targets.

The implications of using angiogenic markers have been progressively increasing. A recent clinical trial by Hofmann et al. [44] investigated the serum level of pro-angiogenic markers in patients diagnosed with MM and non-progressing MGUS. The study identified the following three angiogenesis markers that were correlated with future progression from MGUS to MM: EGF, HGF and angiopoietins-2. These composite angiogenic biomarkers are a potential stratified risk of MGUS progression to MM, which can be added to the established guidelines to improve risk stratification models for MGUS patients. Additionally, high FGF-2 and VEGF plasma levels were negative prognostic markers and were associated with a lower OS in MM patients receiving treatment [45]. More clinical studies encompassing a larger sample size and more angiogenesis-related signals are encouraged to enhance the sensitivity and specificity of these markers.

### 3.2. microRNAs

MicroRNAs (miRNAs) are small non-coding RNAs composed of 18–25 nucleotides that regulate the gene expression via targeting the mRNA affecting various biological processes such as cell proliferation, migration and apoptosis. For decades, researchers have characterized the involvement of miRNAs in the development and progression of various malignancies [46,47,48,49]. They may act as tumor oncogenes or tumor suppressor genes to either enhance or impede tumor growth, respectively. We have previously elaborated the potential role of miRNAs in the diagnosis and prognosis of MM [50]. MiRNAs can potentially serve as molecular biomarkers for MM due to the variable miRNAs detected at different stages of the diseases, therefore providing insights into the diagnosis/prognosis of MM patients [51,52,53,54,55]. Furthermore, miRNAs can be detected in a variety of body fluids, such as plasma, serum and urine, along with being stable for several hours, even at room temperature, thus employing the feasibility and advantage of utilizing non-invasive procedures for miRNAs’ characterization [56,57]. Table 1 summarizes a few miRNAs that can be utilized as diagnostic/prognostic biomarkers in MM.

Some of the well-characterized miRNAs that can distinguish MM-free control from MM and MGUS cases with specificity and sensitivity between 80% and 90% are the up-regulated miR-34a and the down-regulated let-7e [78]. Similarly, high levels of miR-125b-5p, miR-29a and miR-4449, as well as low expressions of miR-30d and miR-203 were reported as potential biomarkers [67,72,79,80,81]. Interestingly, particular miRNAs, such as miR-125b-5p, were specifically detected in the more advanced extramedullary phase of MM [67].

In addition to their diagnostic potentials, miRNAs showed prognostic significance in MM. For instance, a low expression level of miR-15a was associated with a markedly shorter progression-free survival (PFS) and OS [59]. Meanwhile, MM patients with high miR-194 showed higher OS [82]. The overexpression of miR-17 and miR-886-5p was linked to a low OS [83]. Likewise, the MM cases revealing low levels of miR-410 and miR-19a experienced a low PFS and OS [76,84]. In contrast, the downregulation of miR-153, miR-490, miR-500 and miR-642 expression was correlated with better event-free survival [55]. In addition to serum or plasma miRNAs, circulatory exosomal miRNAs such as let-7b and miR-18a possess a prognostic role in MM by predicting the progression of the disease [85].

### 3.3. Telomeres and Activity of Telomerase

Telomeres are nucleoprotein structures that exist at the end of chromosomes and are crucial for protecting chromosomes from degradation [86]. To ensure chromosomal stability and integrity, telomeres form a cap at the chromosome ends, protecting the ends of chromosome arms from inappropriate DNA repair mechanisms and preventing the degradation of genes near the chromosome ends [86]. Telomerase is a ribonucleoprotein complex that serves as a template for the addition of telomeric repeats onto the chromosome ends [87]. Telomeres shorten with every round of cell division and the cells will undergo senescence and apoptosis once the telomeres reach a critical length reduction, thus limiting the proliferation and differentiation of various types of cells [88]. However, in malignant cells, shortened telomeres do not stimulate senescence. In fact, altered telomeres’ architecture and physical properties such as telomerase reverse transcriptase (TERT) mutations, rearrangements and TERT promoter methylation were found to contribute to maintaining malignant cell proliferation and immortalization [89,90]. All such events suggested a significant role of telomere maintenance in unlimited cell proliferation and the tumorigenesis of malignancies [91].

MM patients have longer telomeres compared to the control [92,93]. In MM, a higher TERT amplification was detected and presented a significant association with poor prognosis of the disease [94]. A recent study by Aline et al. compared, in a longitudinal prospective study, the 3D telomeric architecture including telomere intensity, numbers, aggregate and nuclear volume in BM samples from patients with MGUS, SMM or MM, with a minimum follow-up of 5 years. Interestingly, alterations of the telomere structure were specific to different phases of MM and correlated with the aggressiveness of the disease [95]. For instance, there was a substantial increase in telomere numbers in MGUS, when compared with MM. Meanwhile, total telomere intensity and nuclear volume were significantly higher in SMM compared with both MGUS and MM [95]. Furthermore, the intensification of all or some of the above-mentioned telomere parameters was correlated with SMM with a high risk of progression and MM with progressive disease [95]. Other studies have linked the risk, outcome, prognosis, disease heterogeneity, cytogenetic status and OS in MM cases with telomerase activity and telomere length [92,96,97], indicating the potential diagnostic and prognostic properties of telomere in MM.

### 3.4. Extracellular Matrix (ECM) Proteins

**Figure 4:**
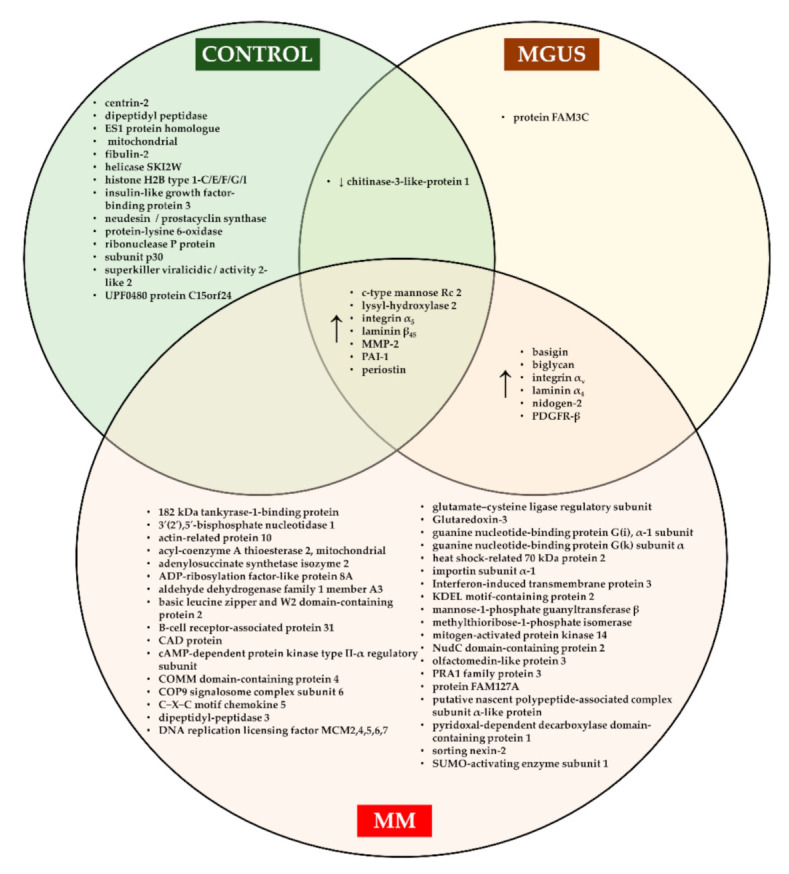
Extracellular matrix proteins that are expressed at different phases of multiple myeloma.

Crucial to MM cell proliferation, prognosis and drug resistance is the interaction between BM microenvironments that consist of cellular and non-cellular components [98]. The cellular part consists of various hematopoietic and non-hematopoietic cells such as stromal cells, endothelial cells, osteoclasts and osteoblasts, while the non-cellular component includes an extracellular matrix (ECM) and other proteins such as cytokines, growth factors and chemokines [98]. In MM, an ECM was found to be remodeled at the gene and protein levels in both MGUS and MM to allow the development of a lenient microenvironment for tumor growth [99]. Various ECM proteins were specifically expressed at different phases of MM, thus representing a potential diagnostic and prognostic tool (Figure 4). For instance, the following ECM proteins: ANXA2 and LGALS1 were expressed more in MM and their abundancy was allied to a decreased OS [99]. Similarly, other proteins associated with cell adhesion, such as laminin subunit beta-1 (encoded by *LAMB1*) and integrin subunit alpha 9 (encoded by *ITGA9*), were dysregulated in MM where both LAMB1 and ITGA9 showed prognostic value and clinical correlation in MM patients [100].

ECM proteins are synthesized by BM stromal cells, which are nonhematopoietic, multipotent progenitor cells that are genetically and functionally altered during the progression of MM [101]. Among BM stromal cells, fibroblasts were shown to support the transition of MGUS to MM [102]. Proteome profiling of BM fibroblasts identified specific proteins acting as biomarkers for the diagnosis/prognosis of MM, where some of these protein markers were characterized to possibly contribute to the transition from MGUS to MM [103]. This may open the door to the development of a risk assessment strategy based on the state of the tumor microenvironment. Though, further research focusing on validating the sensitivity and specificity of these markers is required.

### 3.5. Circulatory Tumor Cells and DNA

Circulatory tumor cells (CTCs) are released into the peripheral circulation from the primary site of the tumor. CTCs were found to exist in MM, representing malignant PCs translocating from the BM to the bloodstream. CTCs are emerging as potential biomarkers for MM, where several studies have reported their prognostic roles in SMM and newly diagnosed and relapsed MM (Table 2). The similarity in genetic profiling between CTCs and malignant PCs in the BM suggested that these markers be used for frequent diagnostic and prognostic evaluations rather than the conventional invasive BM biopsies. For instance, deep sequencing of matched BM and peripheral blood samples of MM cases showed that MM cells were located in the peripheral blood of 96% of the patients, and there was a direct link between the myeloma clone levels in the peripheral blood and the BM [104]. Moreover, 100% of the clonal mutations in the BM samples were verified in CTCs, and 99% of the clonal mutations in CTCs were observed in the BM samples, according to an investigation of eight cases of matched BM and peripheral blood samples [105].

The sequencing of protein-coding exons, e.g., KRAS, BRAF and PIK3CA, in circulating tumor DNA (ctDNA) samples from MM patients revealed that it predicted 96% of mutations detected in matched BM-derived tumor DNA samples with >98% specificity [113]. Likewise, paired BM and ctDNA samples from 48 MM cases showed that out of 128 mutations noted in MM, 38 mutations were observed in both the BM and ctDNA samples. Meanwhile, 59 mutations were found in the BM only [114]. The compatibility found in CTCs and BM cells supports the utilization of CTCs as a replacement for BM biopsy. Though, there are still some considerable discrepancies in the genetic profiles of the BM sample and peripheral CTC populations induced by the heterogeneous character of MM, which requires more clinical investigations to enhance CTCs’ sensitivity as a MM biomarker.

Circulating DNA was shown to significantly increase in the peripheral circulation in several pathological conditions, including cancer. The DNA can be found in either a free form, i.e., cell-free DNA (cfDNA), or in the form of ctDNA. CfDNA is usually released from the cancer cells, thereby possessing the same genetic alterations in the primary tumor cells. The utilization of cfDNA as a prognostic tool in MM has been investigated [115]. The increase in cfDNA was correlated with markedly poor OS. On the other hand, low levels of cfDNA following chemotherapy were associated with an enhanced PFS [116]. Additionally, combining both CTCs and cfDNA analysis can be complementary in replacing BM biopsies to track clonal heterogeneity in MM [117].

### 3.6. Genomic Markers

Cytogenic alternations were included in the revised version of the ISS (Figure 3), indicating their significance in MM assessment. Several other chromosomal translocations and mutations were further reported that can be utilized as biomarkers in MM. These genetic alterations were found to be associated with chemotherapy-induced peripheral neuropathy [118], correlating with the outcome and survival of MM patients [119,120,121,122,123,124,125] and indicating the clinical response to treatment [126,127].

### 3.7. Proteomic Markers

Deregulation of protein expression was reported in MM patients compared to non-malignant samples, demonstrating their diagnostic and/or prognostic potentials. Several studies showed a differential expression of various proteins such as haptoglobin, kininogen 1, transferrin, serum amyloid A protein, plasma kallikrein, integrin alpha-11, apolipoprotein A-I, sulfhydryl oxidase 1 and isoform-1 of multimerin-1 in the sera of MM patients [128,129,130].

Interestingly, the up-regulation of proteins associated with protein folding and proteasome functions, including proteasome activator complex subunit 1, heat shock protein 90, stress-induced-phosphoprotein 1 and protein disulfide-isomerase, was correlated with refractory response bortezomib-based therapy [131], whereas the proteins functioning during inflammation and apoptosis were down-regulated in patients not responding to bortezomib-based therapy [132]. The up-regulation of other proteins, such as Zinc-a-2-glycoprotein, amyloid-A protein and vitamin D-binding protein, was found to play a role in the prediction of a thalidomide response in MM patients [133]. Collectively, the proteomic markers exhibited useful potentials as diagnostic and prognostic tools. However, they still need systematic validation to be deployed in clinical settings.

### 3.8. Immunological Markers

The introduction of several immune-based approaches in treating MM has been revolutionized over the years to become one of the most investigated areas for novel MM therapeutics. Recently approved drugs such as elotuzumab [134] and daratumumab [135], in addition to other immunomodulatory therapies under investigation, including chimeric antigen receptor (CAR) T-cell therapy, are showing great therapeutic potential. Though, there is still a need to validate these and other therapeutic agents in order to have consistency in their effectiveness across a wide range of patients. To achieve this, efficient predictive immune biomarkers are required. Yet, few immune markers have been validated in MM. These markers include CD38, CD55 and CD59. The pretreatment expression of CD38 was correlated with a better outcome to daratumumab, while lack of response and tumor progression was associated with expression of both CD55 and CD59 [136]. Additionally, high-density neutrophils isolated from the peripheral blood of newly diagnosed MM patients showed up-regulation of CD64 and down-regulation of CD16, which were associated with increased immune-suppression [137]. Furthermore, high CD64 in MM patients receiving bortezomib, thalidomide and dexamethasone were associated with an inferior median OS [137]. Table 3 summarizes the results of a few studies that examined the value of immune biomarkers as potential prognostic tools in MM.

Future potential immune biomarkers in MM include neoantigens, a group of antigenic products of somatic mutations that are specific to tumors [149]. The tumor-specific neoantigens do not induce autoimmune toxicity, therefore, they are utilized as drug targets for cancer immunotherapies based on the continuously reported neoantigen-specific T cell responses [150,151]. Although a positive association between neoantigens and clinical responses in several cancers has been reported, the potential role of neoantigens in MM as prognostic biomarkers is still lacking. A recent study suggested high somatic mutation and neoantigen burden were correlated with decreased PFS in MM patients [152]. Additionally, relapsed MM patients demonstrated an increased neoantigen load compared to newly diagnosed patients [153]. Furthermore, the majority of the neoantigens identified by Perumal et al. [153] were not shared between patients and are highly patient-specific. Therefore, future studies are necessary to characterize ideal immune markers to efficiently identify the patients who would ideally benefit from immunotherapy.

## 4. Advanced Methodologies in Next-Generation Biomarkers

In addition to exploring new markers, clinicians and researchers are further conserving the utilization of emerging diagnostic/prognostic techniques and procedures that can be utilized in managing MM (Table 4).

### 4.1. Flow Cytometry

Due to its unique properties in terms of cell analysis, flow cytometry (FC) has swiftly moved from fundamental research to clinical facilities. Flow cytometry technology is currently used in cancer research for a variety of purposes, including the identification of tumor cells, DNA aneuploidy, analysis of cell proliferation and immunophenotyping [154]. In MM, specific markers were utilized to determine the remission status following MM treatment via assessing the minimal residual disease (MRD). The magnitude of the treatment response is commonly evaluated based on paraprotein levels representing residual disease that may contribute to relapse. The PFS and OS were longer in newly diagnosed MM patients with negative MRD detected using FC, suggesting FC as a powerful prognostic tool [155]. Similarly, treated MM cases without MRD after ASCT had a superior PFS and OS compared to MRD positive cases [168]. Interestingly, MRD was shown to be an independent factor predicting complete remission in patients after therapy [169,170]. The more advanced next-generation flow cytometry using EuroFlow can detect low levels of MRD that help in sensitive MRD monitoring and more efficient predictive capabilities [171,172,173,174].

### 4.2. Next-Generation Sequencing (NGS)

With the development of advanced sequencing technology, next-generation sequencing (NGS) has been broadly applied in clinical oncology in the last decade. NGS is extremely helpful when it comes to identifying novel cancer mutations and characterizing molecular rationale for targeted therapy. Unlike traditional sequencing, NGS can fully sequence almost all mutations for thousands of genes at a lower cost. Yet, there are still some challenges that must be overcome for easier utilization of NGS in cancer management. More flexible throughput, as well as feasible data analysis and interpretation, are potential areas of improvement. NGS has been used in the determination of MRD in MM [175,176,177]. Negative MRD detected by deep sequencing revealed a significantly longer progression time (80 months versus 31 months) and a superior OS (not reached versus 81 months) compared to MRD positive. Likewise, using NGS, patients with less than 10−6 tumor cells showed a three-year PFS at 83% versus 53% for patients with more than 10−6 cells at lenalidomide maintenance following chemotherapy [178]. In newly diagnosed MM and SMM patients treated with chemotherapy followed by lenalidomide, the 18 months PFS was markedly different in MRD-negative and MRD-positive patients (100% versus 84%, respectively) [179]. NGS played a significant role in determining MM mutational heterogenicity indicated by the variety of mutated genes and subclonality observed in myeloma disease, thus supporting the importance of developing case-specific and individualized treatment plans for MM cases.

### 4.3. Liquid or Blood Biopsy

As we have highlighted earlier the increasing significance of CTCs and cfDNA in the diagnosis and prognosis of MM in Section 3.5, collecting these biomarkers from peripheral circulation is achieved via liquid biopsy. It represents a promising novel minimally invasive technique for the detection of disease phases and progression when compared to BM biopsy. Even though BM biopsy is frequently used in MM cases, it still represents a burden due to the associated severe pain, tissue injury and potential biopsy errors resulting in it being not applicable for frequent utilization for continuous and proper disease monitoring as well as accurate assessment. Therefore, a liquid biopsy could represent a superior technique due to the high similarity, highlighted earlier in this review, between both procedures with regard to myeloma clone levels and clonal mutations [105]. Moreover, a liquid biopsy is less invasive, and its possible frequent applications could account for MM clonal heterogeneity that might be missed by a single BM biopsy.

### 4.4. Allele-Specific Oligonucleotide qPCR

MRD can be further assessed with high sensitivity using an allele-specific oligonucleotide qPCR (ASO-qPCR) via detecting specific IgH gene rearrangements in MM PCs [172]. A remarkably higher number of tumor cells was detected using an ASO-qPCR in samples from MM patients with progressive disease compared with patients in remission [167]. An ASO-qPCR could further be used in evaluating tumor burden and PFS subsequent to a BM transplant [180]. When comparing both FC and an ASO-qPCR for the assessment of MRD, a substantial correlation was observed in MRD quantitation in both techniques [181]. Among 170 MM patients, the cases with higher MRD showed a markedly lower PFS and OS [181]. Despite the feasible and easy applicability of an ASO-qPCR, it is only applicable to about 42% of MM cases.

## 5. Conclusions and Future Directions

For decades, conventional biomarkers such as M protein, β2 microglobulin, albumin, cytogenic alterations and bone lesions assessed using serum protein electrophoresis, FISH and imaging were used to diagnose and risk-stratify MM. These markers are currently established and incorporated in diagnosis and staging guidelines. Though, the rise in the number of MM cases further expanded the research exploring and developing better diagnostic or prognostic biomarkers. The next-generation biomarkers, including miRNAs, angiogenic markers, ECM proteins, telomeres and telomerase activity have shown great potential in MM diagnosis and prognosis. Moreover, competent immune markers are now emerging to enhance immunotherapy effectiveness via promoting predictive values and customizing treatment plans following proper assessment. Likewise, the utilization of new methodologies in MM diagnosis and risk assessment is critical. In this review, we discussed the significance of employing flow cytometry, NGS, ASO-qPCR and blood biopsy as novel diagnostic and prognostic procedures in MM. Blood biopsy, for instance, could examine the serum levels of CTCs, miRNAs and cfDNA in the peripheral circulation, which provide an advantageous early, frequent and less painful assessment of the disease status compared to the invasive BM biopsy. Although the novel biomarkers show great potential, they have been studied in a small cohort of MM cases. Therefore, for them to be adopted in clinical settings, thorough evaluation and validation in more clinical trials with a large sample size are still required.

## Figures and Tables

**Table 1:** MiRNAs acting as potential diagnostic and/or prognostic biomarkers in MM and their molecular targets.

miRNA	Expression	Potential Molecular Pathways	Ref.
miR-15a and miR-16a	↓	Regulate proliferation and growth of MM cells in vitro and in vivo via inhibiting AKT serine/threonine-protein-kinase (AKT3), ribosomal-protein-S6, MAP-kinases and NF-κB-activator MAP3KIP3	[58]
miR-16	↓	BMSCs-induced overproduction of IL-6 reduces miR-16 expression, thus enhancing cellular proliferation and drug resistance	[59,60]
miR-17	↑	Targets PTEN, E2F1 and Bcl2l11/BIM pathways, thereby enhancing tumor growth	[61,62]
miR-19b	↓	Controls proliferation of cancer stem cells by regulating the TSC1/mTOR signaling pathway	[63,64]
miR-25	↓	Regulates TNF-related apoptosis-inducing ligand (TRAIL)-induced cell death	[64,65]
miR-92	↑	Targets PTEN, E2F1 and Bcl2l11/BIM pathways, thereby enhancing tumor growth	[61,62]
miR-92a	↑	Induces time-dependent down-regulation of c-jun	[64,66]
miR-125b-5p	↑	Inhibited PHLPP2, leading to elevated Akt signaling	[67,68]
miR-129	↓	Regulate the expression of MAP3K7, a kinase able to activate NF-κB circuit to enhance cell proliferation and cycle procession and hinder apoptosis	[69]
miR-153	↓	Regulates the expression of BCL2 and MCL1	[55,70]
miR-194	↓	Increases the therapeutic action of MDM2 inhibitors in vitro and in vivo by enhancing their p53-activating effects	[71]
miR-203	↓	Regulates the Jagged1-Notch1 signaling pathway	[72,73]
miR-296	↓	Targets high-motility group At-hook gene 1 (HMGA1) protein leading to a decrease in cell proliferation and invasion	[55,74]
miR-373	↑	Enhances cell migration and invasion in vitro and in vivo by suppression of CD44	[55,75]
miR-410	↑	Targets KLF10 via activating PTEN/PI3K/AKT pathway, thus enhancing cell proliferation, cell cycle progression and apoptosis inhibition in both in vitro and in vivo	[76]
miR-500	↓	Inhibits cellular proliferation, migration, invasion and adhesion and enhances cells apoptosis	[55,77]

↑ up-regulated; ↓ down-regulated; MAP, mitogen-activated protein; NF-κB, nuclear factor kappa-light-chain-enhancer of activated B cells; BMSCs, bone marrow stromal cells; IL, interleukin; miR, microRNA; PTEN, phosphatase and tensin homolog; E2F1, E2F Transcription Factor 1; TSC1, Tuberous sclerosis proteins 1; mTOR, mechanistic target of rapamycin; MDM2, mouse double minute 2 homolog; PI3K, Phosphoinositide 3-kinase; PHLPP2, PH domain and leucine rich repeat protein phosphatase 2.

**Table 2:** Circulatory tumor cells and their potential prognostic role in SMM and newly diagnosed and relapsed MM.

Study Features	Level/Parameter	Diagnostic/Prognostic Value	Ref.
91 SMM patients at risk of progression	PCs > 5000 × 10^6^/L	- ↑ progression to active disease within two years of diagnosis compared to controls - ↓ median time to progression from 57 to 12 months independent of M protein levels - ↓ OS from 148 to 49 months	[106]
100 SMM patients	≥150 circulating PCs	- Circulating PCs had 78% positive predictive value of progression to MM with 97% specificity - Median time to progression of 9 months compared to not reached for patients with ≤150 CTCs	[107]
157 newly diagnosed MM patients	≥400 circulating PCs	- ↑ PCs proliferation and adverse cytogenetics - ↓ Median OS from 26 to 14 months - ↓ Median time-to-next-treatment to 32 months from not reached in patients with <400 circulating PCs - ↑ ISS stage, creatinine and BM PC percentage	[108]
225 newly diagnosed MM elderly patients	Percentage of differentiated PCs	PFS and OS were low with less and intermediate differentiation stages of PCs	[109]
647 previously treated MM patients	≥100 circulating PCs	- In patients with plateaued disease, ↓ median survival to 22 months from not reached - In patients with active relapsing disease, ↓ median survival from 33 to 12 months	[108]
42 relapsed and refractory MM patients	Presence of pretreatment circulating PCs	- ↓ median time for progression from 456 to 218 days - No difference in OS	[110]
Patients undergoing ASCT	Presence of circulating PCs	- ↓ median PFS from 29.6 to 15.1 months - ↓ median OS to 41 months from not reached	[111]
264 newly diagnosed plasma cell neoplasms patients	Presence of circulating PCs	- Higher numbers of CTCs were associated with higher levels of BM infiltration, more adverse prognostic features, shorter time for MGUS to MM progression and shorter survival	[112]

↑ upregulated; ↓ downregulated; PCs, plasma cells; MM, multiple myeloma; SMM, smoldering multiple myeloma; CTCs, circulatory tumor cells; OS, overall survival; BM, bone marrow; PFS, progression free survival; ASCT, autologous stem cell transplantation.

**Table 3:** Immune biomarkers and their prognostic role in MM.

Study Features	Immune Biomarker	Prognostic Value	Ref.
65 MM and cancer-free cases	MCP-3, VEGF, FGF-2 and TGF-α	Low levels of these biomarkers were detected among future MM patients and increased risk of progression	[138]
268 B-cell lymphomas patients (including 76 MM patients)	MCP-3, FGF-2, TGF-α, MIP-1α, VEGF, fractalkine	Biomarkers showed an inverse association with risk of MM	[139]
177 MM patients undergone ASCT from 2007 to 2016	oligoclonal immunoglobulin bands, i.e., clonal isotype switch (CIS)	- CIS after ASCT was correlated with increased PFS to 52.2 from 36.6 months and OS to 75.1 from 65.4 months - Different isotype of CIS was found in relapsed patients - CIS was associated with decreased CD8 T-cell percentages and a higher CD4/CD8 ratio	[140]
372 newly diagnosed MM patients	ALC to AMC ratio in the peripheral blood	ALC/AMC ≥ 3.6 was associated with superior PFS (43 versus 24 months) and OS (62 versus 48 months) compared with ALC/AMC < 3.6	[141]
201 newly diagnosed MM patients	LMR and u-Ig levels	ORR and OS were decreased in cases with LMR <3.6 and u-Ig decreased by ≥2 items	[142]
285 newly diagnosed MM patients	LMR levels	- Patients with LMR ≤4.2 had poorer OS and PFS than those with LMR > 4.2 - LMR less than 4.2 is an independent predictor for the OS and PFS	[143]
130 MM patients receiving Mel200 and ASCT	ALC, AMC, ANC, LMR, NLR and Ig	- Low ALC and AMC had a low TFS (18 versus 23 months) and (13 versus 25 months), respectively - Low LMR had a decreased TFS (16 versus 52 months) - Patients with two or three suppressed Ig levels had low TFS (17 versus 51 months) - Poor (low LMR and 2–3 suppressed Ig) and good (high LMR and 0–1 suppressed Ig) risk groups showed a median TFS of 7.5 versus 79 months, respectively	[144]
150 MM patients treated with BCD	(neutrophils + monocytes)/lymphocytes ratio (NMLR)	- Low NMLR was associated with decreased β2-microglobulin, serum creatinine and calcium and increased partial response - Low NMLR was correlated with a superior median PFS (24.0 versus 15.5 months) - NMLR was an independent predictor of PFS including non-high-risk cytogenetics	[145]
102 newly diagnosed MM patients	ALC and LMR	- ALC <1.43 × 109/L and LMR <3.7 predicted shorter OS - ALC and LMR were independent predictors for OS	[146]
45 MM stage I (MMI) and 50 MM stage III (MMIII)	IKZF1 and IKZF3 of T-cells	High IKZF3, but not IKZF1, correlates with superior OS in MMIII treated with immunomodulatory drugs	[147]
685 progressing or stable MGUS patients	Serum protein and monoclonal Ig, free light chains and light chains	Progressive MGUS was associated with IgA, >15 g/L monoclonal spike, skewed (<0.1 or >10) serum free light chains ratio	[148]

MCP-3, monocyte chemotactic protein-3; MIP-1 α, macrophage inflammatory protein-1 alpha; VEGF, vascular endothelial growth factor; FGF-2, fibroblast growth factor-2; TGF-α, transforming growth factor-alpha; ASCT, autologous stem cell transplantation; PFS, progression-free survival; OS, overall survival; ALC, absolute lymphocyte count; AMC, absolute monocyte count; LMR, lymphocyte-to-monocyte ratio; u-Ig, uninvolved immunoglobulin; ANC, absolute neutrophil count; NLR, neutrophil to lymphocyte ratio; TFS, treatment-free survival; Ig, immunoglobulin; BCD, bortezomib, cyclophosphamide and dexamethasone; ORR, objective response rate.

**Table 4:** Advanced methodologies and their potential roles in diagnosis and prognosis of MM.

Method/Technique	Features	Diagnostic/Prognostic Value	Ref.
Multiparameter Flow Cytometry (MPC)	Panels of fluorochrome-conjugated antibodies with distinct fluorescence excitation and emission characteristics bind specifically to particular cell phenotypes. Fluorochromes become excited by different lasers to define a high-content molecular signature for each cell	- Assessing MRD to determine the remission status following MM treatment	[154,155,156,157]
		- Quantification of clonal PCs via MPC characterizes patients with SMM at a very high risk of progression to MM within two years	[107]
		- Immunophenotyping of MM patients can help identifying patients at a high risk of progression via detection of CD44, CD45 and CD28 expression and absence of CD117	[158]
Next-generation sequencing (NGS)	A reversible-terminator-based sequencing that can read up to 300 bps with paired-end sequencing. NGS can identify chimeric DNA molecules where the two ends originate from different chromosomes of chromosomal segments, e.g., a translocation breakpoint	- Characterizing almost all mutations associated with case-specific MM development to identify a molecular rationale for targeted therapy	[159]
		- Identifying missense protein-coding alterations in MM cases to rapidly determine risk groups	[160]
		- Identifying certain mutations such as *TP53* that are associated with progressive disease in newly diagnosed MM patients	[161]
		- Sequencing of ctDNA can determine sub-clonal hierarchies for MM profiling, which was found to be highly similar to that of BM samples	[113]
Liquid/blood biopsy	A novel minimally invasive technique for characterizing MM phases and progression when compared to BM biopsy via detecting biomarkers in the peripheral circulation	- Identifying circulating extracellular vehicles (EVs) to be used for monitoring disease burden, disease progression and development of MDR in MM	[160]
		- Detecting CTCs in peripheral blood of MM cases can help in risk stratifying and early diagnosis of MM	[162]
		- Qualitative assessment of cytogenetic alterations found in ctDNA may help in evaluating overtime clonal evolution of MM	[163]
		- Analyzing EVs content was shown to have a prognostic value and might predict drug resistance in MM cases	[85,164]
Allele-specific oligonucleotide (ASO)-qPCR	A short oligonucleotide complementary to sequence of a variable target DNA. It is usually labeled with a radioactive, enzymatic or fluorescent tag. It is highly sensitive to detect a difference of as little as one base in the target’s sequence	- A sequential analysis of MRD in BM samples from MM patients quantified residual MM cells in patients who were considered to be in complete remission	[165]
		- Detecting MRD in autografts of ASCT patients was associated with lower PFS	[166]
		- Measuring tumor load in BM at 3–6 months post-high dose therapy helped in determining patients who are in need of subsequent treatment autologous transplantation	[167]

PCs, plasma cells; MM, multiple myeloma; CTCs, circulatory tumor cells; BM, bone marrow; PFS, progression-free survival; ASCT, autologous stem cell transplantation; MRD, minimal residual disease; ctDNA, circulatory DNA.
